# Zwitterionic 1-{(1*E*)-[(4-hy­droxy­phen­yl)iminio]meth­yl}naphthalen-2-olate: crystal structure and Hirshfeld surface analysis

**DOI:** 10.1107/S205698901701458X

**Published:** 2017-10-20

**Authors:** Bhai R. Devika, C. R. Girija, Suresh Shalini, Mukesh M. Jotani, Edward R. T. Tiekink

**Affiliations:** a Research & Development Centre, Bharathiar University, Coimbatore 641 046, India; b Govt. Science College, Nrupathunga Road, Bangalore 560 001, India; c SSMRV College, Jayanagar 4th T block, Bangalore 560 041, India; d Department of Physics, Bhavan’s Sheth R. A. College of Science, Ahmedabad, Gujarat 380 001, India; e Research Centre for Crystalline Materials, School of Science and Technology, Sunway University, 47500 Bandar Sunway, Selangor Darul Ehsan, Malaysia

**Keywords:** crystal structure, zwitterion, Schiff base, hydrogen bonding, Hirshfeld surface analysis

## Abstract

The title zwitterion, C_17_H_13_NO_2_ (systematic name: 1-{(1*E*)-[(4-hy­droxy­phen­yl)iminium­yl]meth­yl}naphthalen-2-olate), features an intra­molecular charge-assisted N^+^—H⋯O^−^ hydrogen bond. A twist in the mol­ecule is evident around the N—C(hy­droxy­benzene) bond [C—N—C—C torsion angle = 39.42 (8)°] and is reflected in the dihedral angle of 39.42 (8)° formed between the aromatic regions of the mol­ecule. In the crystal, zigzag supra­molecular chains along the *a* axis are formed by charge-assisted hy­droxy-O—H⋯O(phenoxide) hydrogen bonding. These are connected into a layer in the *ab* plane by charge-assisted hy­droxy­benzene-C—H⋯O(phenoxide) inter­actions and π–π contacts [inter-centroid distance between naphthyl-C_6_ rings = 3.4905 (12) Å]. Layers stack along the *c* axis with no specific inter­actions between them. The Hirshfeld surface analysis points to the significance C⋯H contacts between layers.

## Abstract 2

The title zwitterion exists in the iminium/phenoxide form. The mol­ecule is twisted around the N—C(benzene) bond with the C=N—C—C torsion angle being 39.42 (8)°. In the crystal, a zigzag supra­molecular chain is sustained by charge-assisted hy­droxy-O—H⋯O(phenoxide) hydrogen bonding.

## Chemical context   

Schiff bases derived from *o*-hy­droxy­naphthalehyde have attracted significant attention owing to their biological properties, such as anti­-tumour activity (Richardson & Bernhardt, 1999 ▸; Gou *et al.*, 2015 ▸), and their photophysical properties, such as thermo- and photochromism (Matijević-Sosa *et al.*, 2006 ▸). Furthermore, the physical properties of these mol­ecules led to their application in various areas of materials science, such as in the control and measurement of radiation intensity, display systems and optical memory devices (Dürr, 1989 ▸; Hadjoudis & Mavridis, 2004 ▸). These Schiff bases have also been used as tools for assessing the nature of hydrogen bonding (Richardson & Bernhardt, 1999 ▸), as well as keto–amine and phenol–imine tautomerism (Ünver *et al.*, 2000 ▸) in related mol­ecules. In view of these various applications, our recent investigations have focused on the structure determination of Schiff bases of this type, *e.g*. of (*E*)-*N*-[(2-meth­oxy­naphthalen-1-yl)methyl­idene]-3-nitro­aniline (Bhai *et al.*, 2015 ▸). As a continuation of these studies, the crystal and mol­ecular structures of the title compound, (I), are described herein along with an analysis of the Hirshfeld surface, performed in order to gain more information on the nature of the mol­ecular packing.

## Structural commentary   

**Figure 1:**
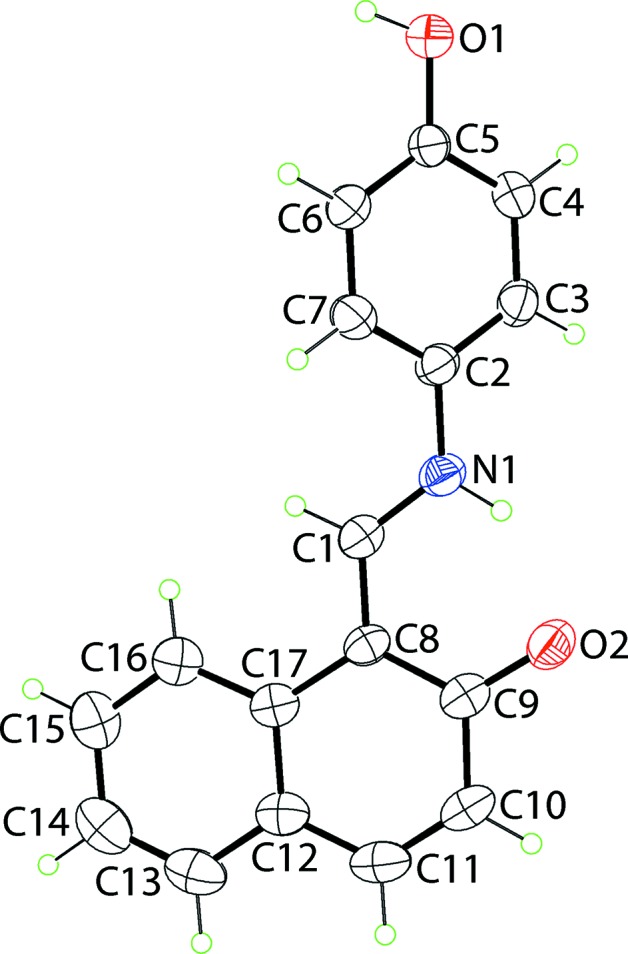
The mol­ecular structure of (I), showing the atom-labelling scheme and displacement ellipsoids at the 35% probability level.

The mol­ecular structure of (I) is shown in Fig. 1 ▸. Crystallography established the mol­ecule to exist in a zwitterionic form with the putative H atom of the naphthyl-hy­droxy group being located on the imine-N atom. This assignment is supported by the short C9—O2 bond length of 1.283 (2) Å. The mol­ecule features two planar regions connected by an imine (iminium­yl) bridge; the configuration about the imine bond [C1=N = 1.308 (2) Å] is *E*. The twist in the mol­ecule occurs around the N1—C2 bond, is seen in the value of the C1—N1—C2—C7 torsion angle of 31.1 (3)°. The dihedral angle between the two aromatic regions is 39.42 (8)°. The coplanar relationship between the imine and naphthyl residues is stabilized by an intra­molecular charge-assisted N^+^—H⋯O^−^ hydrogen bond, Table 1 ▸.
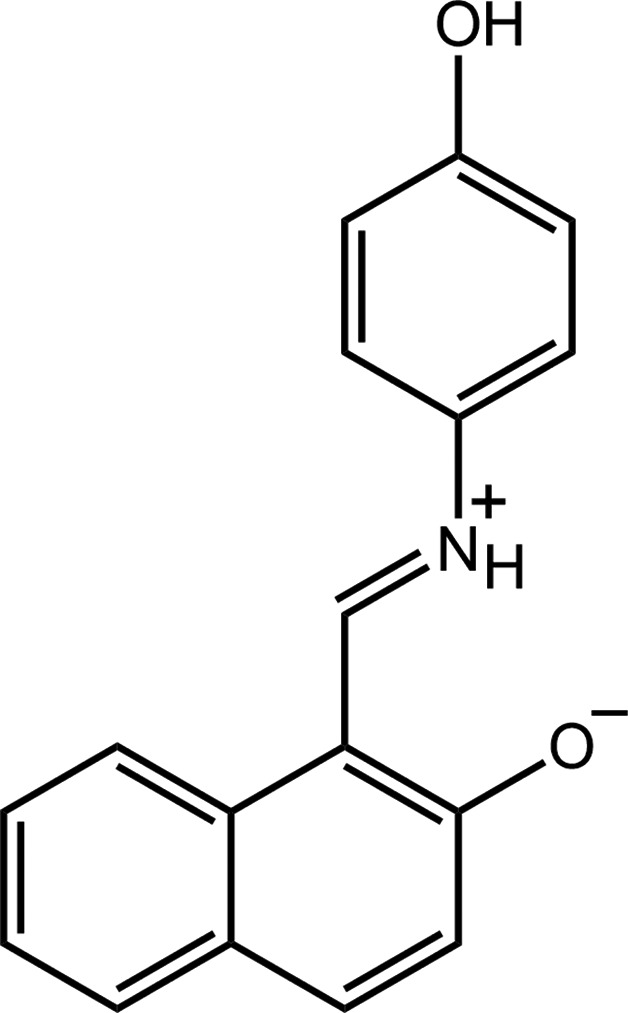


## Supra­molecular features   

**Figure 2:**
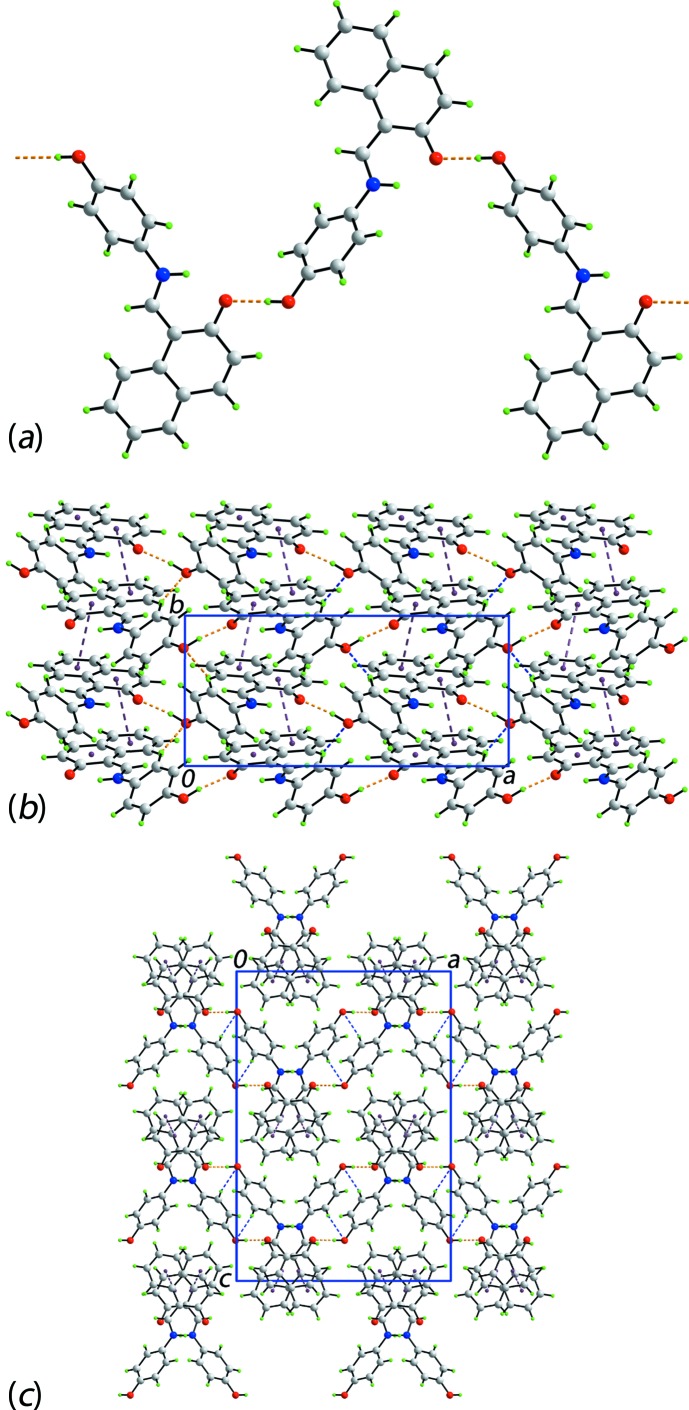
The mol­ecular packing for (I): (*a*) a view of the zigzag supra­molecular chain along the *a* axis mediated by charge-assisted hy­droxy-O—H⋯O(phenoxide) hydrogen bonding, (*b*) a view of the supra­molecular layer in the *ab* plane stabilized by charge-assisted hy­droxy­benzene-C—H⋯O(phenoxide) inter­actions and π–π contacts and (*c*) a view of the unit-cell contents shown in projection down the *b* axis, highlighting the stacking of layers along the *c* axis. The O—H⋯O, C—H⋯O and π–π inter­actions are shown as orange, blue and purple dashed lines, respectively.

The most prominent feature of the mol­ecular packing is the formation of a zigzag (glide symmetry) supra­molecular chain along the *a* axis mediated by hy­droxy-O—H⋯O(phenoxide) charge-assisted hydrogen bonding, Fig. 2 ▸ *a* and Table 1 ▸. Chains are connected into a supra­molecular layer in the *ab* plane by charge-assisted hy­droxy­benzene-C—H⋯O(phenoxide) inter­actions, Table 1 ▸, as well as π–π contacts between the two rings of the naphthyl residue; the inter-centroid separation for (C8–C12,C17)⋯(C12–C17)^i^ = 3.4905 (12) Å and angle of inclination = 2.68 (8)° [symmetry code (i) 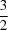 − *x*, 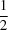 + *y*, *z*], Fig. 2 ▸ *b*. Layers stack along the *c* axis with no directional inter­actions between them, Fig. 2 ▸ *c*.

## Analysis of the Hirshfeld surface   

**Figure 3:**
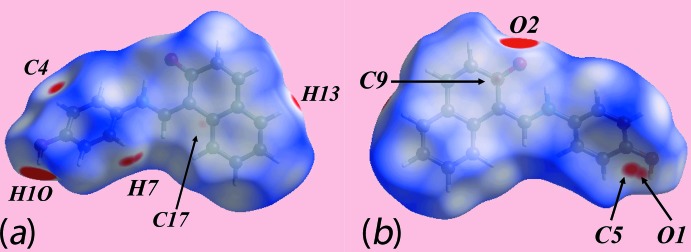
Views of the Hirshfeld surface for (I) mapped over *d*_norm_ in the range −0.150 to +1.460 a.u.

**Figure 4:**
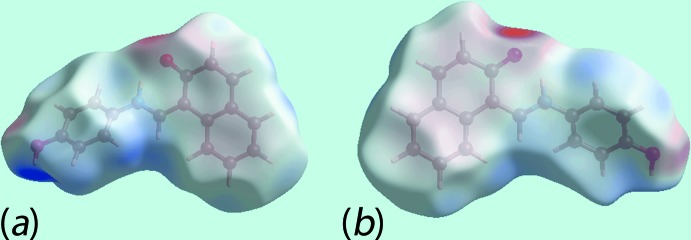
Views of the Hirshfeld surface for (I) mapped over the electrostatic potential in the range ±0.116 a.u.

**Figure 5:**
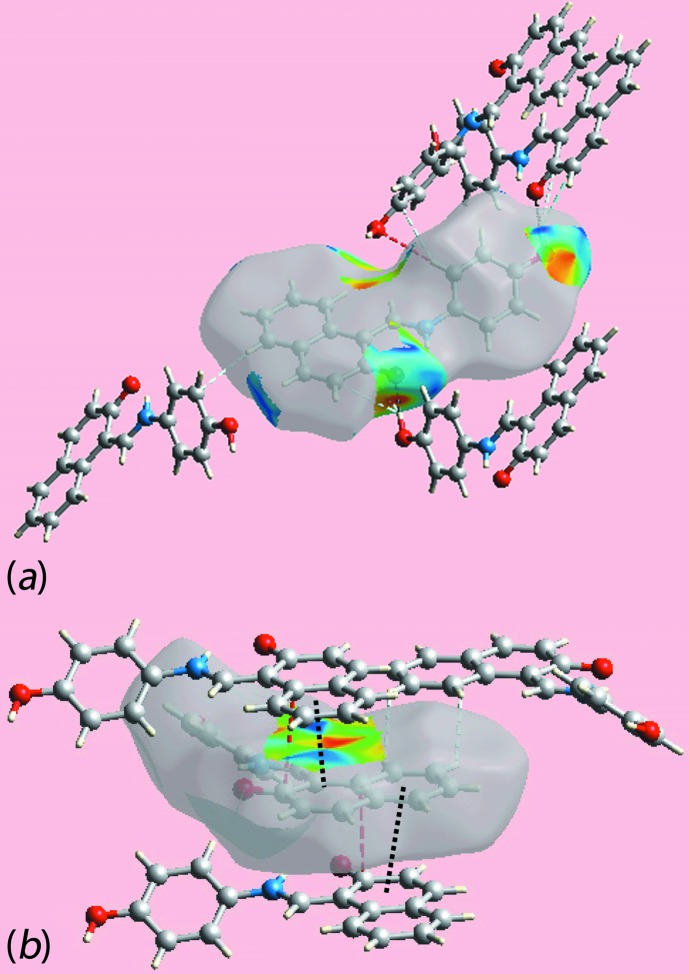
Views of the Hirshfeld surfaces about a reference mol­ecule mapped over the shape-index property highlighting (*a*) inter­molecular O—H⋯O and C—H⋯O inter­actions, and short inter­atomic C⋯H/H⋯C contacts by black, red and sky-blue dashed lines, respectively, and (*b*) short inter­atomic C⋯C and H⋯H contacts, and π–π stacking inter­actions by red, sky-blue and black dashed lines, respectively.

The Hirshfeld surface was calculated for (I) according to earlier work on organic mol­ecules (Tan *et al.*, 2017 ▸) and provides more detailed information on the inter­molecular inter­actions influential in the crystal. In addition to the bright-red spots near those atoms participating in charge-assisted O1—H1O⋯O2 and C7—H7⋯O1 inter­actions on the Hirshfeld surface mapped over *d*_norm_, Fig. 3 ▸, the bright-red spots appearing near the benzene-C4, -C5 and -H7, and naphthyl-H13 atoms are indicative of short inter­atomic C⋯H/H⋯C contacts significant in the crystal, Table 2 ▸. The C4⋯H13 contact occurs in the inter-layer region. Further, the short inter­atomic C⋯C contacts between the naphthyl-C9 and -C17 atoms, Table 2 ▸, assigned to π–π stacking inter­actions, appear as faint-red spots in Fig. 3 ▸. The donors and acceptors of the aforementioned inter­actions appear as blue and red regions, respectively, around the atoms on the Hirshfeld surface mapped over electrostatic potential in the views shown in Fig. 4 ▸. The short inter­atomic contacts together with the charge-assisted O—H⋯O and C—H⋯O inter­actions formed with the atoms of a reference mol­ecule within shape-index mapped Hirshfeld surface are highlighted in the views of Fig. 5 ▸.

**Figure 6:**
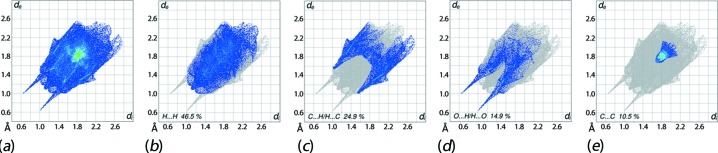
(*a*) The full two-dimensional fingerprint plots for (I), and those delineated into (*b*) H⋯H, (*c*) C⋯H/H⋯C, (*d*) O⋯H/H⋯O and (*e*) C⋯C contacts.

The overall two-dimensional fingerprint plot, Fig. 6 ▸ *a*, and those delineated into H⋯H, C⋯H/H⋯C, O⋯H/H⋯O and C⋯C contacts (McKinnon *et al.*, 2007 ▸) are illustrated in Figs. 6 ▸ *b*–*e*, respectively; the relative contributions from different inter­atomic contacts to the Hirshfeld surfaces are summarized in Table 3 ▸. The presence of a small peak in the centre at *d*_e_ + *d*_i_ ∼ 2.3 Å in the fingerprint plot delineated into H⋯H contacts, Fig. 6 ▸ *b*, results from the short inter­atomic H⋯H contact between symmetry related naphthyl-H15 and -H16 atoms, Table 2 ▸. In the fingerprint plot delineated into C⋯H/H⋯C contacts, Fig. 6 ▸ *c*, the short inter­atomic contacts summarized in Table 2 ▸ appear as the points distributed as the pair of thick spikes with tips at *d*_e_ + *d*_i_ ∼ 2.6 Å. The presence of charge-assisted O—H⋯O hydrogen bonds in the structure are characterized by the distinctive spikes with tips at *d*_e_ + *d*_i_ ∼ 1.7 Å, Fig. 6 ▸ *d*, whereas the points belong to inter­molecular C—H⋯O hydrogen bond are merged within the plot. The fingerprint plot delineated into C⋯C contacts, Fig. 6 ▸ *e*, indicate the presence of the π–π stacking inter­actions between symmetry related naphthyl-(C8–C12/C17) and -(C12–C17) rings through the arrow-shaped distribution with the green points spread about *d*_e_ = *d*_i_ = 1.8 Å. The small contributions from other inter­atomic contacts summarized in Table 3 ▸ have negligible effect on the mol­ecular packing.

## Database survey   

The most closely related structure to (I) in the crystallographic literature (Groom *et al.*, 2016 ▸) is that of the ethanol hemisolvate of (I), *i.e*. (I)·0.5EtOH (Safia *et al.*, 2015 ▸). Here, there are two mol­ecules in the asymmetric unit and each exists in the zwitterionic form with C—O^−^ = 1.288 (4) and 1.2943 (19) Å. By contrast to (I), the zwitterions in (I)·0.5EtOH are more planar than in (I), with the dihedral angles between the aromatic residues being 7.59 (4)° in one of the independent zwitterions and 8.01 (4)° in the other. The other structure deserving of comment is that of 2-{[(4-hy­droxy­phen­yl)imino]­meth­yl}phenol, where the 2-oxidonaphthyl group of (I) has been replaced by a 2-oxido­benzene residue. This has been crystallized in two forms, *viz.* a *P*2_1_/*c* form with *Z*′ = 2 (Ersanlı *et al.*, 2004 ▸) and a *C*2/*c* form with *Z*′ = 1 (Wang *et al.*, 2011 ▸). The common feature of the three mol­ecules is the formation of hydrox­yl/imine tautomer, as opposed to zwitterionic (I) and (I)·0.5EtOH (Safia *et al.*, 2015 ▸). The three mol­ecules have smaller deviations from planarity than (I), as seen in the dihedral angles between the aromatic rings of 10.43 (6) and 15.70 (6)° for the *P*2_1_/*c* form, and 14.91 (9)° for the *C*2/*c* form. Finally, a deprotonated form of (I), with the 4-hy­droxy group intact, forms a six-membered {Pd—O—C C—C=N} chelate ring in its bis-complex with palladium(II) (Tardiff *et al.*, 2007 ▸).

## Synthesis and crystallization   

4-Hy­droxy­aniline (0.00916 mol, 1.00 g) was added to a solution of 2-hy­droxy-1-napthaldehyde (0.00916 mol, 1.58 g) in ethanol (25 ml). The resulting mixture was refluxed at 333 K and stirred for 2.5 h. The reaction mixture was cooled to room temperature and the resulting orange precipitate was filtered off and washed with cold ethanol to obtain the pure product in 65% yield. Crystals of (I) were grown from a mixture of chloro­form and methanol (1:1 *v*/*v*) by slow evaporation.

## Refinement   

Crystal data, data collection and structure refinement details are summarized in Table 4 ▸. The carbon-bound H atoms were placed in calculated positions (C—H = 0.95 Å) and were included in the refinement in the riding-model approximation, with *U*_iso_(H) values set at 1.2*U*_eq_(C). The O- and N-bound H atoms were located in a difference Fourier map, but were refined with distance restraints of O—H = 0.82±0.01 Å and N—H = 0.86±0.01 Å, and with *U*_iso_(H) values set at 1.5*U*_eq_(O) and 1.2*U*_eq_(N), respectively. To confirm the positions of the acidic-H atoms, a separate refinement was conducted whereby no distance restraints were applied resulting in O—H and N—H bond lengths of 0.93 (2) and 1.00 (3) Å, respectively.

## Supplementary Material

Crystal structure: contains datablock(s) I, global. DOI: 10.1107/S205698901701458X/ex2001sup1.cif

Structure factors: contains datablock(s) I. DOI: 10.1107/S205698901701458X/ex2001Isup2.hkl

CCDC reference: 1429885

Additional supporting information:  crystallographic information; 3D view; checkCIF report

## Figures and Tables

**Table 1:** Hydrogen-bond geometry (Å, °)

*D*—H⋯*A*	*D*—H	H⋯*A*	*D*⋯*A*	*D*—H⋯*A*
N1—H1N⋯O2	0.88 (1)	1.81 (2)	2.553 (2)	141 (2)
O1—H1O⋯O2^i^	0.89 (1)	1.74 (1)	2.622 (2)	173 (1)
C7—H7⋯O1^ii^	0.93	2.60	3.487 (3)	160

Symmetry codes: (i) 
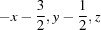
; (ii) 
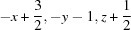
.

**Table 2:** Summary of short inter­atomic contacts (Å) in (I)

Contact	Distance	Symmetry operation
C4⋯H13	2.69	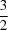 − *x*, − *y*, 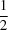 + *z*
C5⋯H7	2.69	1 − *x*, 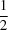 + *y*, 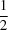 − *z*
C9⋯H1O	2.638 (16)	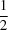 + *x*, *y*, 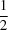 − *z*
C9⋯C17	3.367 (2)	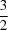 − *x*, − 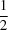 + *y*, *z*
H15⋯H16	2.38	1 − *x*, − *y*, − *z*

**Table 3:** Percentage contributions of inter­atomic contacts to the Hirshfeld surface for (I)

Contact	Percentage contribution
H⋯H	46.5
C⋯H/H⋯C	24.9
O⋯H/H⋯O	14.9
C⋯C	10.5
C⋯O/O⋯C	1.0
N⋯H/H⋯N	1.0
N⋯O /O⋯N	0.6
C⋯N/N⋯C	0.6

**Table 4:** Experimental details

Crystal data	
Chemical formula	C_17_H_13_NO_2_
*M*_r_	263.28
Crystal system, space group	Orthorhombic, *P* *b* *c* *a*
Temperature (K)	293
*a*, *b*, *c* (Å)	15.7473 (14), 7.3042 (5), 22.7257 (19)
*V* (Å^3^)	2613.9 (4)
*Z*	8
Radiation type	Mo *K*α
μ (mm^−1^)	0.09
Crystal size (mm)	0.35 × 0.25 × 0.10

Data collection	
Diffractometer	Bruker Kappa APEXII CCD
Absorption correction	Multi-scan (*SADABS*; Bruker, 2016 ▸)
*T*_min_, *T*_max_	0.941, 0.982
No. of measured, independent and observed [*I* > 2σ(*I*)] reflections	17744, 2253, 1518
*R*_int_	0.043
(sin θ/λ)_max_ (Å^−1^)	0.591

Refinement	
*R*[*F* ^2^ > 2σ(*F* ^2^)], *wR*(*F* ^2^), *S*	0.041, 0.115, 1.04
No. of reflections	2253
No. of parameters	189
No. of restraints	2
H-atom treatment	H atoms treated by a mixture of independent and constrained refinement
Δρ_max_, Δρ_min_ (e Å^−3^)	0.15, −0.16

Computer programs: *APEX3* and *SAINT* (Bruker, 2016 ▸), *SHELXS97* (Sheldrick, 2008 ▸), *SHELXL2014* (Sheldrick, 2015 ▸), *ORTEP-3 for Windows* (Farrugia, 2012 ▸), *DIAMOND* (Brandenburg, 2006 ▸) and *publCIF* (Westrip, 2010 ▸).

## supplementary crystallographic information

### Crystal data

**Table:**

C_17_H_13_NO_2_	*D*_x_ = 1.338 Mg m^−^^3^
*M**_r_* = 263.28	Mo *K*α radiation, λ = 0.71073 Å
Orthorhombic, *P**b**c**a*	Cell parameters from 3652 reflections
*a* = 15.7473 (14) Å	θ = 2.5–22.5°
*b* = 7.3042 (5) Å	µ = 0.09 mm^−^^1^
*c* = 22.7257 (19) Å	*T* = 293 K
*V* = 2613.9 (4) Å^3^	Block, colourless
*Z* = 8	0.35 × 0.25 × 0.10 mm
*F*(000) = 1104	

### Data collection

**Table:**

Bruker AXS Kappa APEXII CCD diffractometer	1518 reflections with *I* > 2σ(*I*)
ω and φ scans	*R*_int_ = 0.043
Absorption correction: multi-scan (SADABS; Bruker, 2016)	θ_max_ = 24.8°, θ_min_ = 2.2°
*T*_min_ = 0.941, *T*_max_ = 0.982	*h* = −17→18
17744 measured reflections	*k* = −8→5
2253 independent reflections	*l* = −26→26

### Refinement

**Table:**

Refinement on *F*^2^	Hydrogen site location: mixed
Least-squares matrix: full	H atoms treated by a mixture of independent and constrained refinement
*R*[*F*^2^ > 2σ(*F*^2^)] = 0.041	*w* = 1/[σ^2^(*F*_o_^2^) + (0.0542*P*)^2^ + 0.3444*P*] where *P* = (*F*_o_^2^ + 2*F*_c_^2^)/3
*wR*(*F*^2^) = 0.115	(Δ/σ)_max_ < 0.001
*S* = 1.04	Δρ_max_ = 0.15 e Å^−^^3^
2253 reflections	Δρ_min_ = −0.16 e Å^−^^3^
189 parameters	Extinction correction: SHELXL2014 (Sheldrick, 2015), Fc^*^=kFc[1+0.001xFc^2^λ^3^/sin(2θ)]^-1/4^
2 restraints	Extinction coefficient: 0.0094 (11)

### Special details

**Table:**

Geometry. All esds (except the esd in the dihedral angle between two l.s. planes) are estimated using the full covariance matrix. The cell esds are taken into account individually in the estimation of esds in distances, angles and torsion angles; correlations between esds in cell parameters are only used when they are defined by crystal symmetry. An approximate (isotropic) treatment of cell esds is used for estimating esds involving l.s. planes.

### Fractional atomic coordinates and isotropic or equivalent isotropic displacement parameters (Å^2^)

**Table:**

	*x*	*y*	*z*	*U*_iso_*/*U*_eq_	
O1	0.50441 (9)	0.2166 (2)	0.36926 (6)	0.0610 (4)	
O2	0.85495 (9)	0.0585 (2)	0.13238 (6)	0.0619 (4)	
N1	0.70553 (11)	0.0841 (2)	0.17521 (7)	0.0508 (4)	
C1	0.68015 (13)	0.0167 (2)	0.12484 (8)	0.0478 (5)	
H1	0.6223	−0.0034	0.1199	0.057*	
C2	0.65339 (12)	0.1142 (3)	0.22458 (8)	0.0466 (5)	
C3	0.67240 (12)	0.2537 (3)	0.26274 (8)	0.0510 (5)	
H3	0.7199	0.3265	0.2562	0.061*	
C4	0.62150 (13)	0.2861 (3)	0.31052 (8)	0.0522 (5)	
H4	0.6342	0.3821	0.3359	0.063*	
C5	0.55214 (12)	0.1784 (3)	0.32124 (7)	0.0471 (5)	
C6	0.53407 (13)	0.0373 (3)	0.28381 (8)	0.0606 (6)	
H62	0.4873	−0.0373	0.2908	0.073*	
C7	0.58493 (14)	0.0058 (3)	0.23593 (9)	0.0617 (6)	
H7	0.5725	−0.0910	0.2108	0.074*	
C8	0.73349 (12)	−0.0267 (2)	0.07838 (7)	0.0436 (5)	
C9	0.82215 (13)	−0.0075 (2)	0.08526 (9)	0.0492 (5)	
C10	0.87583 (14)	−0.0632 (3)	0.03806 (10)	0.0588 (6)	
H10	0.9345	−0.0557	0.0423	0.071*	
C11	0.84314 (15)	−0.1261 (3)	−0.01218 (10)	0.0643 (6)	
H11	0.8801	−0.1621	−0.0419	0.077*	
C12	0.75490 (14)	−0.1402 (3)	−0.02208 (8)	0.0533 (5)	
C13	0.72247 (18)	−0.1984 (3)	−0.07628 (10)	0.0704 (7)	
H13	0.7599	−0.2305	−0.1062	0.084*	
C14	0.63811 (19)	−0.2091 (3)	−0.08611 (10)	0.0742 (7)	
H14	0.6176	−0.2476	−0.1224	0.089*	
C15	0.58281 (16)	−0.1625 (3)	−0.04171 (10)	0.0679 (6)	
H15	0.5246	−0.1697	−0.0482	0.082*	
C16	0.61235 (14)	−0.1058 (3)	0.01170 (9)	0.0571 (5)	
H16	0.5737	−0.0761	0.0411	0.069*	
C17	0.69907 (13)	−0.0913 (2)	0.02326 (8)	0.0460 (5)	
H1O	0.4563 (9)	0.154 (2)	0.3685 (9)	0.069*	
H1N	0.7609 (7)	0.095 (3)	0.1766 (9)	0.082 (8)*	

### Atomic displacement parameters (Å^2^)

**Table:**

	*U*^11^	*U*^22^	*U*^33^	*U*^12^	*U*^13^	*U*^23^
O1	0.0498 (9)	0.0847 (11)	0.0486 (8)	−0.0037 (7)	0.0043 (7)	−0.0102 (7)
O2	0.0443 (9)	0.0836 (10)	0.0577 (9)	0.0018 (7)	0.0002 (7)	0.0086 (7)
N1	0.0434 (11)	0.0608 (11)	0.0482 (10)	−0.0001 (8)	0.0060 (8)	0.0034 (7)
C1	0.0427 (12)	0.0513 (11)	0.0493 (12)	0.0016 (9)	0.0034 (9)	0.0052 (9)
C2	0.0397 (11)	0.0558 (11)	0.0444 (11)	−0.0003 (9)	0.0040 (9)	0.0028 (9)
C3	0.0431 (12)	0.0576 (12)	0.0524 (12)	−0.0095 (9)	−0.0018 (9)	0.0015 (9)
C4	0.0519 (13)	0.0567 (12)	0.0480 (11)	−0.0030 (10)	−0.0056 (9)	−0.0067 (9)
C5	0.0394 (12)	0.0626 (12)	0.0392 (10)	0.0029 (9)	−0.0030 (9)	−0.0021 (8)
C6	0.0510 (13)	0.0763 (14)	0.0546 (12)	−0.0212 (11)	0.0115 (10)	−0.0124 (11)
C7	0.0611 (15)	0.0691 (14)	0.0550 (13)	−0.0174 (11)	0.0104 (10)	−0.0177 (10)
C8	0.0409 (12)	0.0443 (10)	0.0455 (10)	0.0046 (8)	0.0055 (9)	0.0082 (8)
C9	0.0467 (12)	0.0484 (11)	0.0527 (12)	0.0055 (9)	0.0060 (10)	0.0132 (9)
C10	0.0441 (13)	0.0581 (13)	0.0743 (15)	0.0068 (10)	0.0167 (11)	0.0091 (11)
C11	0.0722 (17)	0.0536 (13)	0.0671 (15)	0.0053 (11)	0.0281 (13)	0.0009 (11)
C12	0.0642 (15)	0.0428 (11)	0.0530 (12)	0.0023 (10)	0.0136 (11)	0.0065 (9)
C13	0.098 (2)	0.0580 (13)	0.0553 (14)	0.0023 (12)	0.0181 (13)	−0.0011 (10)
C14	0.104 (2)	0.0611 (14)	0.0575 (14)	−0.0012 (13)	−0.0080 (15)	−0.0014 (11)
C15	0.0748 (17)	0.0584 (14)	0.0706 (15)	0.0020 (11)	−0.0147 (13)	0.0028 (11)
C16	0.0608 (15)	0.0538 (12)	0.0568 (13)	0.0057 (10)	−0.0004 (11)	0.0038 (10)
C17	0.0520 (13)	0.0366 (10)	0.0495 (11)	0.0049 (8)	0.0053 (9)	0.0085 (8)

### Geometric parameters (Å, º)

**Table:**

O1—C5	1.354 (2)	C8—C9	1.412 (3)
O1—H1O	0.886 (10)	C8—C17	1.444 (3)
O2—C9	1.283 (2)	C9—C10	1.425 (3)
N1—C1	1.308 (2)	C10—C11	1.334 (3)
N1—C2	1.408 (2)	C10—H10	0.9300
N1—H1N	0.877 (10)	C11—C12	1.411 (3)
C1—C8	1.386 (2)	C11—H11	0.9300
C1—H1	0.9300	C12—C13	1.400 (3)
C2—C7	1.362 (3)	C12—C17	1.401 (3)
C2—C3	1.371 (2)	C13—C14	1.349 (3)
C3—C4	1.370 (3)	C13—H13	0.9300
C3—H3	0.9300	C14—C15	1.376 (3)
C4—C5	1.368 (3)	C14—H14	0.9300
C4—H4	0.9300	C15—C16	1.364 (3)
C5—C6	1.366 (2)	C15—H15	0.9300
C6—C7	1.371 (3)	C16—C17	1.395 (3)
C6—H62	0.9300	C16—H16	0.9300
C7—H7	0.9300		
			
C5—O1—H1O	110.6 (13)	O2—C9—C8	121.87 (17)
C1—N1—C2	125.31 (18)	O2—C9—C10	119.78 (19)
C1—N1—H1N	111.7 (15)	C8—C9—C10	118.35 (19)
C2—N1—H1N	122.5 (15)	C11—C10—C9	120.9 (2)
N1—C1—C8	124.56 (19)	C11—C10—H10	119.5
N1—C1—H1	117.7	C9—C10—H10	119.5
C8—C1—H1	117.7	C10—C11—C12	122.8 (2)
C7—C2—C3	119.01 (17)	C10—C11—H11	118.6
C7—C2—N1	121.44 (17)	C12—C11—H11	118.6
C3—C2—N1	119.55 (17)	C13—C12—C17	119.7 (2)
C4—C3—C2	120.11 (18)	C13—C12—C11	121.5 (2)
C4—C3—H3	119.9	C17—C12—C11	118.81 (19)
C2—C3—H3	119.9	C14—C13—C12	121.5 (2)
C5—C4—C3	120.62 (18)	C14—C13—H13	119.3
C5—C4—H4	119.7	C12—C13—H13	119.3
C3—C4—H4	119.7	C13—C14—C15	119.2 (2)
O1—C5—C6	122.77 (18)	C13—C14—H14	120.4
O1—C5—C4	117.95 (17)	C15—C14—H14	120.4
C6—C5—C4	119.28 (17)	C16—C15—C14	120.8 (2)
C5—C6—C7	119.95 (19)	C16—C15—H15	119.6
C5—C6—H62	120.0	C14—C15—H15	119.6
C7—C6—H62	120.0	C15—C16—C17	121.6 (2)
C2—C7—C6	121.01 (18)	C15—C16—H16	119.2
C2—C7—H7	119.5	C17—C16—H16	119.2
C6—C7—H7	119.5	C16—C17—C12	117.17 (19)
C1—C8—C9	119.49 (18)	C16—C17—C8	123.76 (17)
C1—C8—C17	120.53 (18)	C12—C17—C8	119.06 (19)
C9—C8—C17	119.98 (17)		
			
C2—N1—C1—C8	−174.22 (17)	C8—C9—C10—C11	2.3 (3)
C1—N1—C2—C7	31.1 (3)	C9—C10—C11—C12	0.5 (3)
C1—N1—C2—C3	−149.93 (18)	C10—C11—C12—C13	176.90 (19)
C7—C2—C3—C4	−1.9 (3)	C10—C11—C12—C17	−1.9 (3)
N1—C2—C3—C4	179.13 (17)	C17—C12—C13—C14	−0.1 (3)
C2—C3—C4—C5	1.0 (3)	C11—C12—C13—C14	−178.9 (2)
C3—C4—C5—O1	179.76 (17)	C12—C13—C14—C15	−0.3 (3)
C3—C4—C5—C6	0.2 (3)	C13—C14—C15—C16	0.1 (3)
O1—C5—C6—C7	−179.99 (19)	C14—C15—C16—C17	0.6 (3)
C4—C5—C6—C7	−0.5 (3)	C15—C16—C17—C12	−0.9 (3)
C3—C2—C7—C6	1.7 (3)	C15—C16—C17—C8	178.00 (18)
N1—C2—C7—C6	−179.39 (19)	C13—C12—C17—C16	0.7 (3)
C5—C6—C7—C2	−0.5 (3)	C11—C12—C17—C16	179.54 (17)
N1—C1—C8—C9	3.4 (3)	C13—C12—C17—C8	−178.30 (16)
N1—C1—C8—C17	−176.69 (16)	C11—C12—C17—C8	0.6 (3)
C1—C8—C9—O2	−3.8 (3)	C1—C8—C17—C16	3.3 (3)
C17—C8—C9—O2	176.27 (16)	C9—C8—C17—C16	−176.73 (17)
C1—C8—C9—C10	176.36 (16)	C1—C8—C17—C12	−177.76 (16)
C17—C8—C9—C10	−3.6 (2)	C9—C8—C17—C12	2.2 (2)
O2—C9—C10—C11	−177.56 (18)		

### Hydrogen-bond geometry (Å, º)

Hydrogen-bond geometry (Å, °) for (I).

**Table:**

*D*—H···*A*	*D*—H	H···*A*	*D*···*A*	*D*—H···*A*
N1—H1*N*···O2	0.88 (1)	1.81 (2)	2.553 (2)	141 (2)
O1—H1*O*···O2^i^	0.89 (1)	1.74 (1)	2.622 (2)	173 (1)
C7—H7···O1^ii^	0.93	2.60	3.487 (3)	160

Symmetry codes: (i) −*x*−3/2, *y*−1/2, *z*; (ii) −*x*+3/2, −*y*−1, *z*+1/2.
